# Nanofibrous bicomponent scaffolds for the dual delivery of NGF and GDNF: controlled release of growth factors and their biological effects

**DOI:** 10.1007/s10856-020-06479-2

**Published:** 2021-01-20

**Authors:** Chaoyu Liu, Xiaohua Li, Qilong Zhao, Yuancai Xie, Xumei Yao, Min Wang, Fengjun Cao

**Affiliations:** 1 Department of Research and Development, Shenzhen Shiningbiotek Co., Ltd, Shenzhen, 518055 P. R. China; 2 Department of Mechanical Engineering, The University of Hong Kong, Pokfulam Road, Hong Kong, P. R. China; 3 Oncology Center, Hubei University of Medicine, Shiyan, 442000 P. R. China; 4 Shenzhen Institutes of Advanced Technology, Chinese Academy of Science, Shenzhen, 518055 P. R. China; 5 Department of Thoracic, Peking University Shenzhen Hospital, Shenzhen, 518036 P. R. China

## Abstract

Electrospun fibrous scaffolds capable of providing dual growth factor delivery in a controlled manner have distinctive advantages for tissue engineering. In this study, we have investigated the formation, structure, and characteristics/properties of fibrous bicomponent scaffolds for the dual delivery of glial cell line-derived neurotrophic factor (GDNF) and nerve growth factor (NGF) for peripheral nerve tissue regeneration. GDNF and NGF were incorporated into core-shell structured poly(lactic-co-glycolic acid) (PLGA) and poly (d,l-lactic acid) (PDLLA) nanofibers, respectively, through emulsion electrospinning. Using dual-source dual-power electrospinning, bicomponent scaffolds composed of GDNF/PLGA fibers and NGF/PDLLA fibers with different fiber component ratios were produced. The structure, properties, and in vitro release behavior of mono- and bicomponent scaffolds were systematically investigated. Concurrent and sustained release of GDNF and NGF from bicomponent scaffolds was achieved and their release profiles could be tuned. In vitro biological investigations were conducted. Rat pheochromocytoma cells were found to attach, spread, and proliferate on all scaffolds. The release of growth factors from scaffolds could induce much improved neurite outgrowth and neural differentiation. GDNF and NGF released from GDNF/PLGA scaffolds and NGF/PDLLA scaffolds, respectively, could induce dose-dependent neural differentiation separately. GDNF and NGF released from bicomponent scaffolds exerted a synergistic effect on promoting neural differentiation.

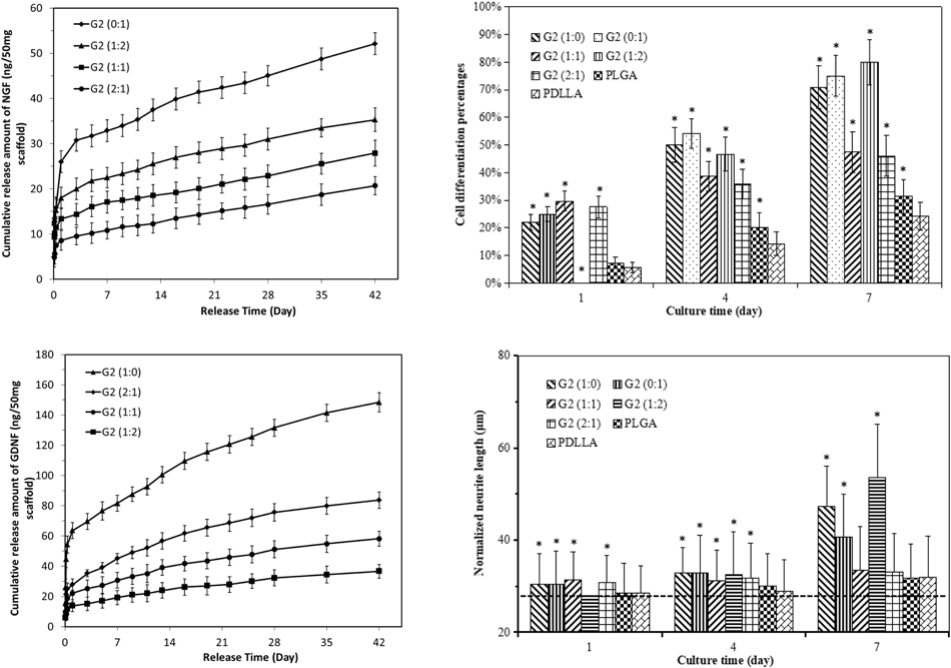

## Introduction

Functional recovery of injured peripheral nerves still needs to be improved in clinic, especially for critical-size peripheral nerve injury. Local delivery of neurotrophic factors including nerve growth factor (NGF) and glial cell line-derived neurotrophic factor (GDNF) at surgical sites have been proposed to enhance peripheral nerve regeneration. These neurotrophic factors play important roles in neuronal survival, neural differentiation, and axonal regeneration independently or synergistically at different effective concentrations [1–5]. Sustained stimulation of neurotrophic factors is also required to induce neural differentiation [6]. However, few studies have been conducted to investigate the influence of dual and sustained neurotrophic factor delivery at biologically effective neurotrophic factor concentrations on neurite outgrowth that may be important for enhanced nerve repair and functional recovery. Suitable delivery vehicles are needed for controlled and sustained delivery of these therapeutic biomolecules due to their vulnerability and short half-life through direct administration [7, 8].

Electrospinning technique is advantageous in producing fibrous scaffolds with micro- to nanoscale architectures resembling extracellular matrix, high surface area to volume ratio, and high porosity. Electrospun fibers with diverse morphology and structures produced by different electrospinning techniques such as emulsion electrospinning and coaxial electrospinning may be utilized as efficient delivery vehicles of various biomolecules for different applications [9, 10]. The rat pheochromocytoma cell line (PC12 cells) is a widely adopted in vitro model for neuronal differentiation. PC12 cells respond to neurotrophic factors including NGF and GDNF and differentiate into neuron-like phenotypes characterized by halted proliferation and neurite outgrowth [6, 11, 12].

In this study, bicomponent fibrous scaffolds were designed and fabricated through dual-source dual-power electrospinning (DSDP-ES) technique established in our previous study [13, 14], with GDNF and NGF being incorporated into PLGA and PDLLA nanofibers, respectively, via emulsion electrospinning. The mass ratios of GDNF/PLGA fibers to NGF/PDLLA fibers in bicomponent scaffolds were varied. Dual and sustained release of GDNF and NGF with different release profiles were achieved from scaffolds with different components. The bioactivities of GDNF and NGF released from GDNF/PLGA fibers and NGF/PDLLA fibers were assessed on a culture-to-culture basis using PC12 cells. The cytotoxicity of PC12 cells to different scaffolds was evaluated. The morphology of cells cultured on scaffolds was examined by SEM and confocal laser scanning microscopy (CLSM). Neurite outgrowth and neural differentiation of PC12 cells on different scaffolds were investigated.

## Materials and methods

### Materials

PLGA (LA:GA = 50:50) and PDLLA with molecular weight of 100 kDa (as indicated by their inherent viscosity 0.6–0.8 dL/g) were purchased from Lakeshore Biomaterials, USA. Chloroform was supplied by Uni Chem Co., Korea. The human β-NGF with Enzyme Linked Immunosorbent Assay (ELISA) Kit, human GDNF with ELISA Kit were purchased from Peprotech Inc. and R&D Systems, Inc., respectively. Dulbecco’s Modified Eagle Medium (DMEM), fetal bovine serum (FBS), penicillin/streptomycin (P/S), and trypsin/EDTA were purchased from Invitrogen, Inc., USA. Mouse anti-neurofilament (M + H) primary antibody, goat anti-mouse IgG secondary antibody (Alexa Fluor^®^ 594), phalloidin (Alexa Fluor^®^ 488), and DAPI (4’, 6-diamidino-2-phenylindole) were purchased from Life Technologies. Triton X-100, Span-80, phosphate buffered saline (PBS) tablets, heparin, bovine serum albumin (BSA), paraformaldehyde, glutaraldehyde, sodium cacodylate, MTT (3-(4,5-dimethylthiazol-2-yl)-2,5-diphenyl tetrazolium bromide), and sucrose were Sigma-Aldrich products. Other chemicals were used as received.

### Fabrication of scaffolds

The fibrous scaffolds incorporated with GFs were fabricated by emulsion electrospinning technique. For water-in-oil (w/o) emulsion formulation, PLGA or PDLLA dissolved in chloroform at a certain concentration was used as the oil phase and NGF or GDNF were dissolved in 0.5 wt% BSA solution as the water phase. The volume ratio of the oil phase to the water phase was fixed at 10:1. 5 wt%. Span-80 (with respect to the weight of polymer used) was added in the polymer solution for the formation and stabilization of emulsions [14]. The oil phase and water phase were mixed for 10 min through magnetic stirring at 300 rpm to form homogeneous w/o emulsions. Polymer concentration was optimized for both PLGA and PDLLA and served as control variable to manipulate fiber diameter. The electrospinning parameters including applied voltage, inner diameter of needle tip, needle-to-collector distance, and feeding rate of emulsions were optimized as 16 kV, 0.8 mm, 8 cm, and 2 mL/h, respectively [14]. To produce fibrous bicomponent scaffolds, DSDP-ES was employed. GDNF/PLGA fibers and NGF/PDLLA fibers were collected simultaneously by a rotating drum collector, forming non-woven bicomponent scaffolds. By using a multiple-syringe strategy, the mass ratio of GDNF/PLGA fibers to NGF/PDLLA fibers in bicomponent scaffolds was adjusted. The formulation of emulsions for producing different mono- and bicomponent scaffolds is summarized in Table 1. The produced electrospun scaffolds were freeze-dried for 24 h before any further experiments.

**Table 1:** Composition of emulsions for producing nonwoven mono- and bicomponent electrospun scaffolds

Scaffold sample	PLGA emulsions		PDLLA emulsions	
	Water phase 1 ml H_2_O	Oil phase 10 ml CHCl_3_	Water phase 1 ml H_2_O	Oil phase 10 ml CHCl_3_
G1 (0:1)	5 µg GDNF 5 mg BSA	1.5 g PLGA 75 µl Span-80	5 µg NGF 5 mg BSA	2 g PDLLA 100 µl Span-80
G1 (1:2)				
G1 (1:1)				
G1 (2:1)				
G1 (1:0)				
G2 (0:1)	4 µg GDNF 5 mg BSA	1.2 g PLGA 60 µl Span-80	3.75 µg NGF 5 mg BSA	1.5 g PDLLA 75 µl Span-80
G2 (1:2)				
G2 (1:1)				
G2 (2:1)				
G2 (1:0)				
PLGA	5 mg BSA	1.5 g PLGA 75 µl Span-80		
PDLLA			5 mg BSA	1.5 g PDLLA 75 µl Span-80

### Characterization of fibers and electrospun scaffolds

Morphological and structural characterization of electrospun fibers and fabricated scaffolds was conducted. Freeze-dried samples of electrospun fibers and scaffolds were sputtered with a thin gold coating for 30 s by a sputter coater (BEL-TACSCD005) and their morphology was examined using SEM (Hitachi S-4800 FEG SEM, Japan). A total of 100 fibers in each scaffold were randomly selected to determine average fiber diameters. Electrospun fibers were also collected during electrospinning on a copper grid covered with carbon film and their structures were observed using TEM (Philips EM208s TEM, the Netherlands).

### In vitro release test of NGF and GDNF

For in vitro release investigations, experiments followed the procedures as used by other research groups [15]. Briefly, each fibrous scaffold with similar thickness was cut into square shape (30 × 30 mm) for testing. Scaffold samples (about 50 mg each) were immersed in 12-well plates filled with 3 mL release medium and incubated in shaking water bath at 37 °C. At pre-set times, 0.4 mL of supernatant was retrieved from each well and replaced by 0.4 mL of fresh release medium. The supernatant sample was frozen at –20 °C for further measurement or directly used for analysis. The concentration of growth factor in the supernatant was determined using ELISA kit. The release medium was prepared by adding 0.5% BSA, 0.05% Tween-20, 0.02% NaN_3_, and 0.1% heparin in PBS solution. At least three replicates were tested for each type of fibrous scaffold and results were expressed as mean ± SD.

### Cell culture

PC12 cells (rat pheochromocytoma cell line, Biowit Technologies, Shenzhen, China) were used for determining the bioactivity of growth factors, cytotoxicity of fibrous scaffolds, and cell proliferation and differentiation on scaffolds. All in vitro experiments used PC12 cells cultured in DMEM culture medium supplemented with glucose (2 g/L), 10% FBS, and 1% P/S antibiotics at 37 °C in humidified air containing 5% CO_2_ unless noted otherwise. When the cells cultured in a flask achieved 80% confluence, they were enzymatically digested by 0.25% (w/v) trypsin-EDTA from the culture flask, and counted using a haemocytometer for further experiments. At least three replicates were tested for each type of fibrous scaffold and results were expressed as mean ± SD.

### Evaluation of cytotoxicity of fibrous scaffolds

In vitro cytotoxicity of fibrous scaffolds was investigated by using MTT assay. Fibrous scaffolds were cut into square shape (0.8 × 0.8 cm), sterilized by ^60^Co γ-irradiation at a dose of 15 kGy and then placed in the wells of 96-well plates. Cells were seeded on the scaffolds at initial density of 4 × 10^3^ cells/well in 200 μL of growth medium and incubated in a 5% CO_2_ humidified atmosphere at 37°C. The culture medium was refreshed every 2 days. Cells cultured in culture medium were used as control. The cytotoxicity at days 1, 4, and 7 was evaluated. In all, 10 μL of MTT solution (5 mg/mL) in PBS was added to each well, and the plate was incubated for 4 h in a 5% CO_2_ humidified atmosphere at 37°C. In all, 100 μL of DMSO was added, and the plates were shaken for 15 min at 37 °C by a shaker incubator to dissolve the formazan crystals. In all, 100 μL of solution in each well was pipetted and transferred to a 96-well plate. The absorbance at 570 nm of each well was measured using a plate reader. The cell viability was calculated using the equation as below:

1 $${\mathrm{Cell}}\,{\mathrm{Viability}}\,\left( \% \right) = {\mathrm{OD}}_{570\left( {\mathrm{sample}} \right)}/{\mathrm{OD}}_{570\left( {\mathrm{control}} \right)} \times 100\% ,$$

where OD_570(sample)_ represented measurement of optical density from the wells loaded with fibrous scaffolds and OD_570 (control)_ from the wells without scaffolds. At least three replicates were tested for each type of fibrous scaffold and results were expressed as mean ± SD.

### Cell proliferation and differentiation on fibrous scaffolds

The cell response on fibrous scaffolds including cell proliferation and differentiation was investigated. Briefly, electrospun scaffold samples with or without growth factors were cut into square shape (2.5 × 2.5 cm, about 5 mg) and sterilized. The scaffold samples were placed in the wells of 12-well culture plates and covered the bottom of each well. About 4 × 10^3^ PC12 cells were seeded on the fibrous scaffolds in each well supplemented with 1 mL culture medium in a 5% CO_2_ humidified atmosphere at 37 °C. The culture medium was refreshed every 2 days. After 1, 4, and 7 days of culture, the cell-scaffold constructs were washed twice with PBS and fixed with 4% PFA for 10 min at room temperature. After washing twice with PBS, the cells were permeabilized with 0.1% Triton X-100 solution and incubated in (1% w/v) BSA block solution for 30 min, followed by the incubation in mouse anti-neurofilament (M + H) primary antibody (1:100 dilution) containing block solution for 1 h. After washing two times with PBS, the cells were incubated with goat anti-mouse IgG secondary antibody (2 drops/mL) for 30 min. FITC phalloidin (1:40 dilution) was simultaneously added for F-actin staining for 30 min and DAPI (1:1000 dilution) was added for nuclei staining for 5 min. Confocal imaging of cells on scaffolds was performed on a confocal microscope (LSM 710, Carl Zeiss).

The morphology of cell-scaffold constructs was studied under SEM. In order to prepare the samples for SEM examination, the cell-scaffold constructs were washed with PBS and subsequently fixed with 2.5% glutaraldehyde at 4 °C for 4 h. After washing with cacodylate buffer containing 0.1 M sucrose, they were further washed with PBS and rinsed with DI water. The cell-scaffold constructs were collected after freeze-drying for 48 h, sputter-coated with a thin layer of gold for 30 s and then observed under SEM. At least three replicates were tested for each type of fibrous scaffold and results were expressed as mean ± SD.

### Image analysis

SEM images were loaded into Image J (National Institute of Health, USA) and 100 fibers in each scaffold were randomly selected to determine average fiber diameters. Confocal microscopic images were loaded into Image J and morphometric data were obtained. The presence of neurites was examined and cell differentiation was quantified by measuring the neurite length. Only protrusions originating from the cell body and longer than 28 μm (about two times of average cell body diameter) were counted as neurites.

## Results

### Morphology and structure of fibrous scaffolds incorporated with growth factors

**Fig. 1:**
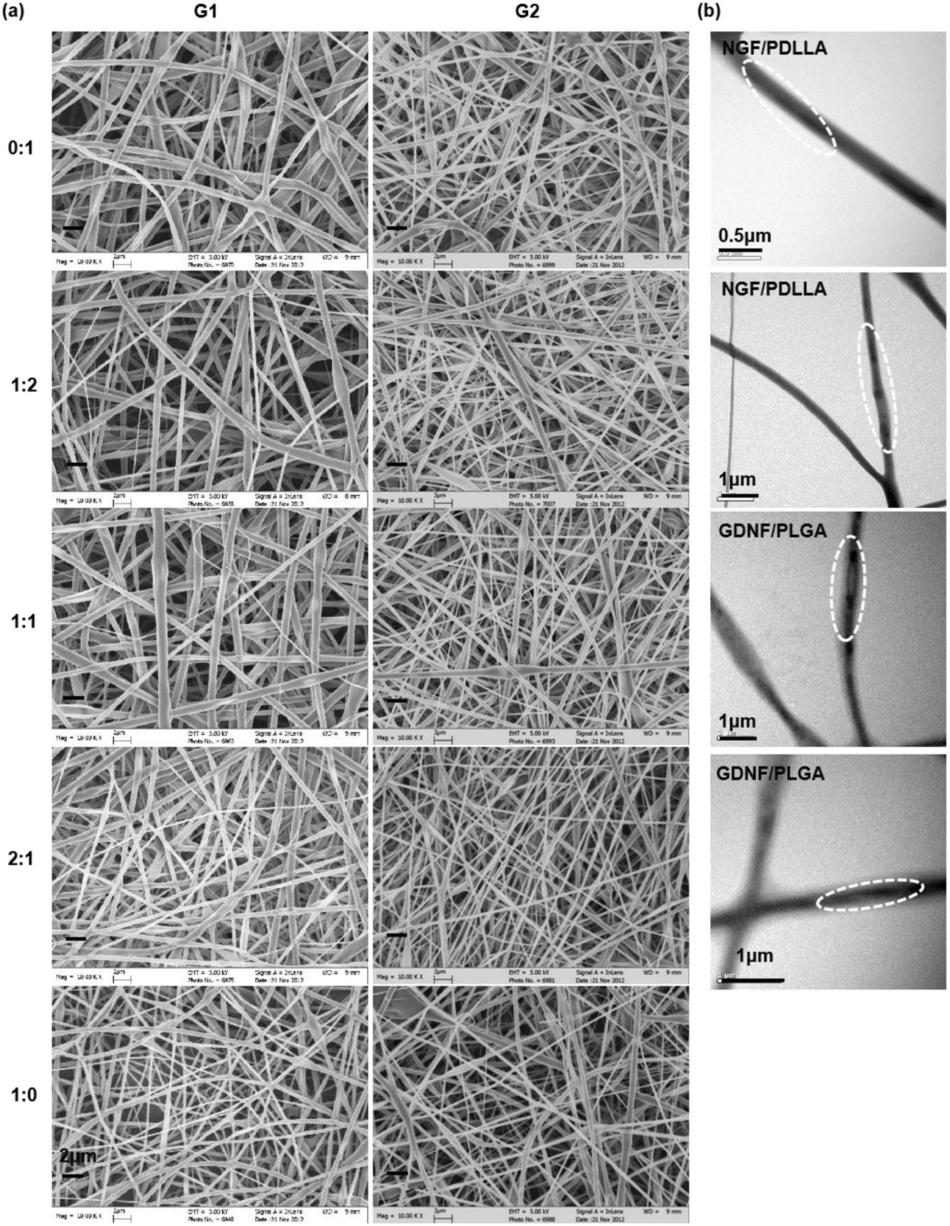
Morphology and structures of electrospun fibers and scaffolds. **a** SEM images of mono- and bicomponent fibrous scaffolds, **b** TEM images of core-shell structured NGF/PDLLA and GDNF/PLGA fibers and discontinuous core-shell structures were noted with dashed ellipse

PLGA and PDLLA-based mono- and bicomponent nanofibrous scaffolds with a thickness of approximate 400 μm were obtained through DSDP-ES technique and emulsion electrospinning. The surface morphology of fibrous scaffolds was examined by SEM and is shown in Fig. 1a. Mono- and bicomponent fibrous scaffolds exhibited non-woven structures of randomly orientated fibers. Porous structure was formed by robust and beadless fibers with smooth surface. The distribution of fibers in bicomponent scaffolds appeared to be random.

Through emulsion electrospinning, core-shell structure was formed in both GDNF/PLGA fibers and NGF/PDLLA fibers, which is shown in Fig. 1b. The water phase core that was enclosed by oil phase shell is marked by dotted ellipse. The core-shell structure existed with discontinuous water phase core in both types of fibers but appeared differently. The phase boundary of core-shell structure in PDLLA fibers was unambiguous, while it appeared in a dispersive pattern in PLGA fibers.

**Fig. 2:**
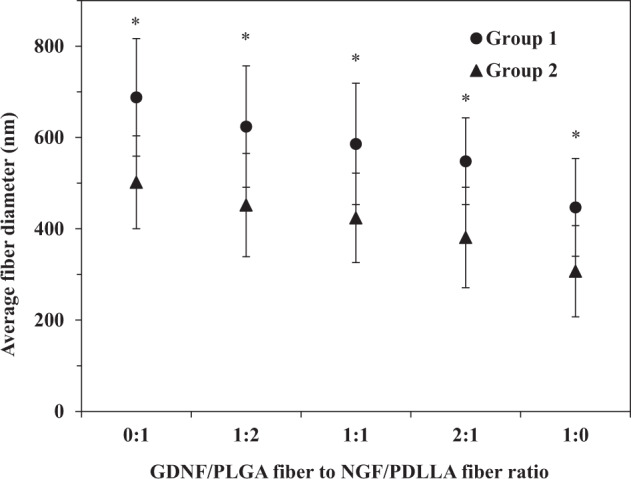
Average fiber diameter in fibrous scaffolds with different fiber component ratios. Fiber diameter in Group 1 statistically different than that in Group 2 for respective fiber component ratios. *p < 0.05

Fiber diameter of various scaffolds was varied by changing polymer concentration during emulsion electrospinning, which was summarized in Fig. 2. The fibrous scaffolds were divided into thick fiber (G1) and thin fiber (G2) groups. The average fiber diameter decreased in both G1 and G2 groups as component ratio of GDNF/PLGA fibers to NGF/PDLLA fibers increased from 0:1 to 1:0.

### In vitro release profiles of NGF and GDNF

**Fig. 3:**
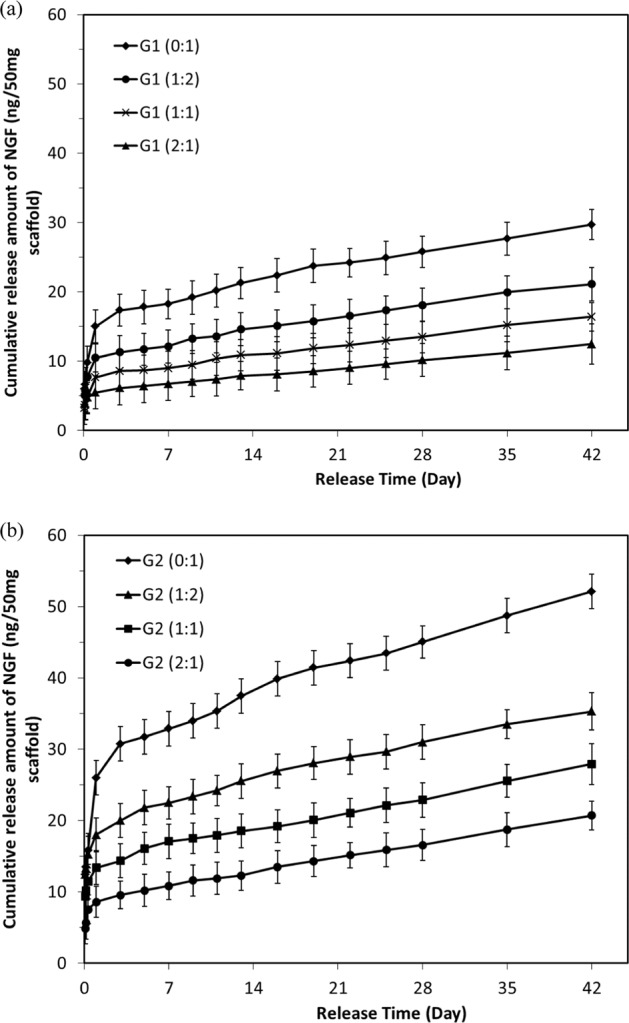
In vitro release of NGF from electrospun scaffolds in 42-day release tests. **a** Group 1, **b** Group 2

**Fig. 4:**
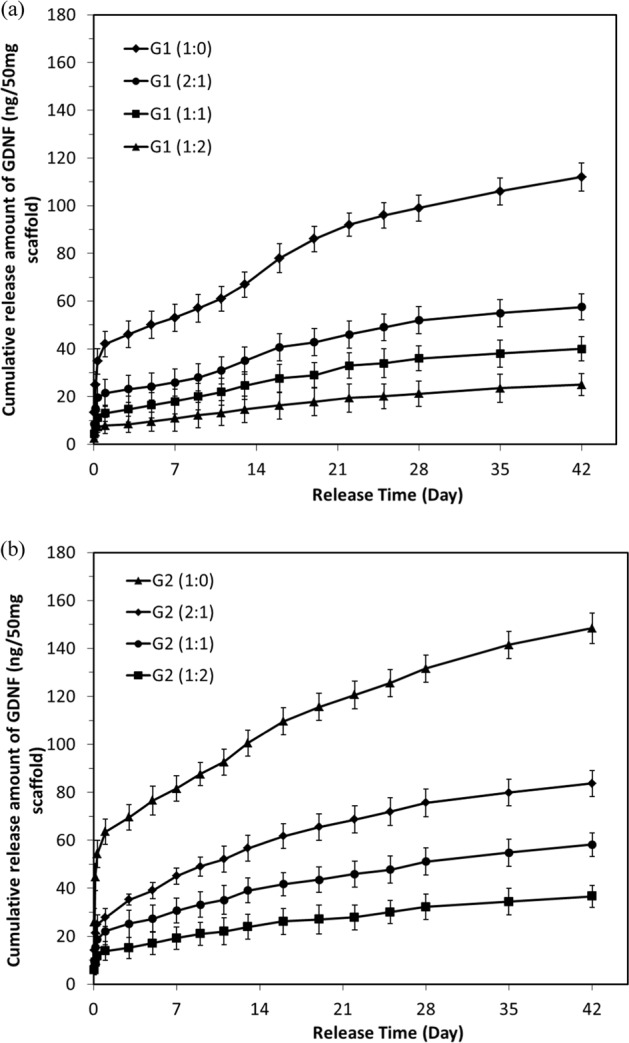
In vitro release of GDNF from electrospun scaffolds in 42-day release tests. **a** Group 1, **b** Group 2

The cumulative release of NGF and GDNF from electrospun scaffolds within a 42-day test are demonstrated in Figs. 3 and 4.

As for the release behavior of NGF, all scaffolds exhibited similar release profile including an initial burst release within 24 h, followed by a much slower and sustained release. After 6-week release, the cumulative release amount of NGF increased to 29.7 ± 2.2, 21.1 ± 2.4, 16.4 ± 2.0, and 12.5 ± 2.9 ng in G1 group and 52.1 ± 2.4, 35.3 ± 2.6, 27.9 ± 2.9, and 20.7 ± 2.0 ng in G2 group, corresponding to GDNF/PLGA fiber to NGF/PDLLA fiber ratio at 0:1, 1:2, 1:1, and 2:1, respectively. The results indicated that cumulative release amount of NGF increased generally with the decrease of GDNF/PLGA fiber to NGF/PDLLA fiber ratio for bicomponent scaffolds. Thinner fiber diameter (G2 group) resulted in more cumulative release amount of growth factors comparing with G1 group at certain GDNF/PLGA fiber to NGF/PDLLA fiber ratio.

A much faster release behavior was observed for GDNF. After an initial burst release within 24 h, a sustained release of GDNF was observed. After 6-week release, the cumulative release amount of GDNF increased to 112.0 ± 6.0, 57.5 ± 5.4, 40.0 ± 5.1, and 25.0 ± 4.6 ng in G1 group and 148.4 ± 6.5, 83.7 ± 5.4, 58.2 ± 4.9, and 36.6 ± 4.6 ng in G2 group, corresponding to GDNF/PLGA fiber to NGF/PDLLA fiber ratio at 1:0, 2:1, 1:1, and 1:2, respectively. The results indicated that cumulative release amount of GDNF from scaffolds increased with the increase of GDNF/PLGA fiber to NGF/PDLLA fiber ratio but with the decrease of fiber diameter. An increase in GNDF release rate was noticed at around 2 weeks of in vitro release, which was particularly obvious in G1 (1:0) scaffolds. The results of in vitro release tests revealed that concurrent and sustained release of NGF and GDNF from bicomponent scaffolds could be successfully achieved using bicomponent scaffolds made by DSDP-ES. The release rate of GDNF was much faster than that of NGF. Thicker fiber diameter could help alleviate the initial burst release and slow down the release rate of both growth factors. Cumulative release amount of NGF and GDNF and their ratios could be tuned by varying GDNF/PLGA fiber to NGF/PDLLA fiber ratios.

### Evaluation of cytotoxicity of fibrous scaffolds

**Fig. 5:**
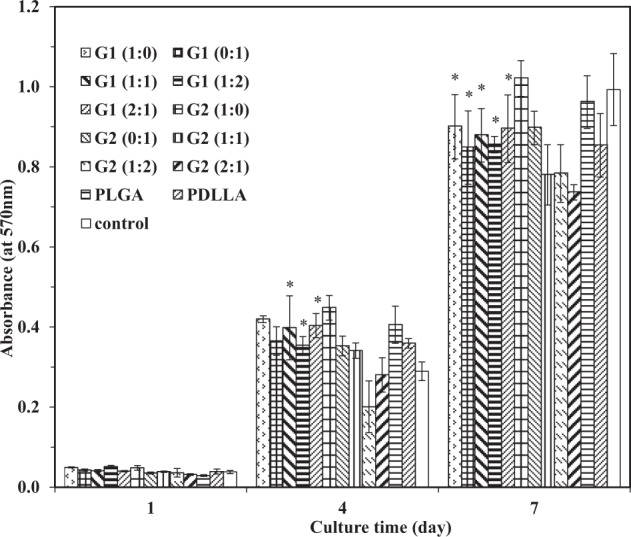
In vitro cytotoxicity assessment of fibrous scaffolds through MTT tests. Cell proliferation in Group 1 statistically different than that in Group 2 for respective fiber component ratios. *p < 0.05

The influence of different scaffolds on cell proliferation was evaluated by MTT method and is shown in Fig. 5. The cell proliferation on different scaffolds and petri dish (control) was more or less the same at day 1. Much improved cell proliferation on scaffolds was observed in all groups at days 4 and 7. At day 4, enhanced cell proliferation was observed on all scaffolds except G2 (1:2) and G2 (2:1) scaffolds as compared to control group. The degree of cell proliferation on PLGA scaffolds was higher as compared to that on PDLLA scaffolds. The highest cell proliferation was found in G2 (1:0) scaffolds and the least proliferation was in G2 (1:2). The cell proliferation on other scaffolds varied with growth factor concentrations and component ratios of GDNF/PLGA fibers to NGF/PDLLA fibers. At day 7, less cell proliferation was observed on all scaffolds except G2 (1:0) scaffolds as compared to control group. The degree of cell proliferation on PLGA scaffolds was higher as compared to that on PDLLA scaffolds. The cell proliferation in different groups was also dependent on growth factor concentrations and component ratios of GDNF/PLGA fibers to NGF/PDLLA fibers. Comparatively lower cell proliferation was observed on bicomponent scaffolds in G2 group that might be attributed to cell differentiation due to growth factors released from scaffolds. All scaffolds exhibited no or negligible cytotoxicity.

### Morphology of PC12 cells on scaffolds

**Fig. 6:**
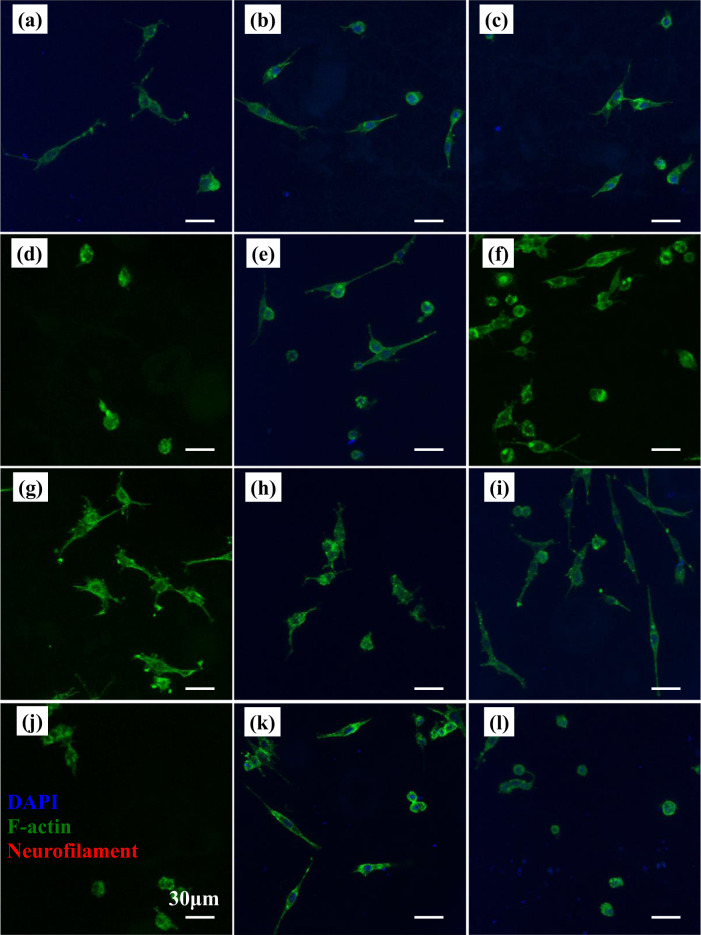
Morphology of PC12 cells cultured on different scaffolds as revealed under a confocal fluorescent microscope at day 1. **a** G1 (1:0) scaffold, **b** G1 (0:1) scaffold, **c** G1 (1:1) scaffold, **d** G1 (1:2) scaffold, **e** G1 (2:1) scaffold, **f** PLGA scaffold, **g** G2 (1:0) scaffold, **h** G2 (0:1) scaffold, **i** G2 (1:1) scaffold, **j** G2 (1:2) scaffold, **k** G2 (2:1) scaffold, and **l** PDLLA scaffold

**Fig. 7:**
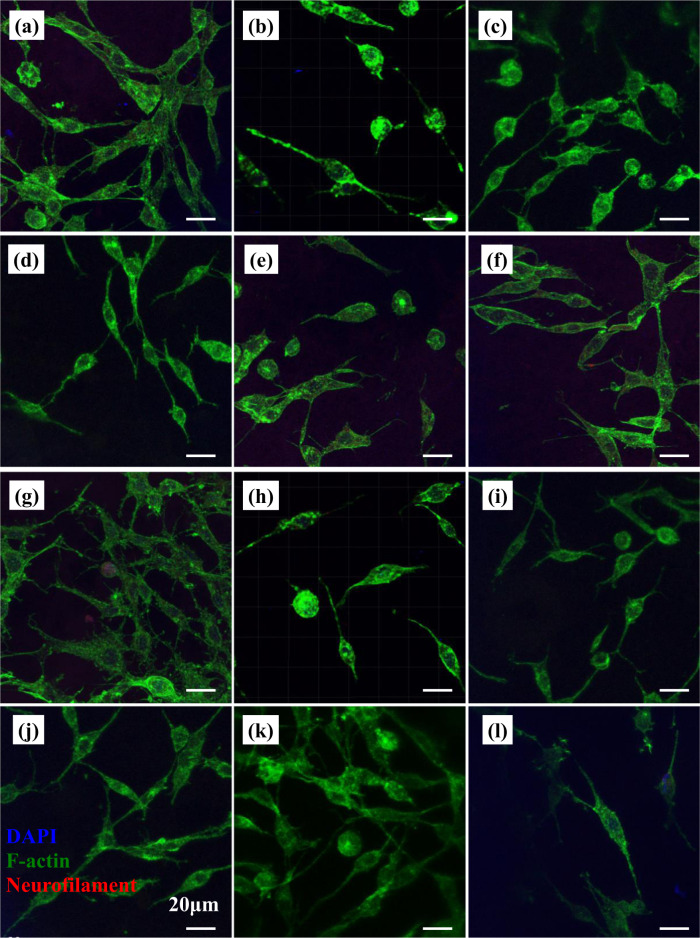
Morphology of PC12 cells cultured on different scaffolds as revealed under a confocal fluorescent microscope at day 4. **a** G1 (1:0) scaffold, **b** G1 (0:1) scaffold, **c** G1 (1:1) scaffold, **d** G1 (1:2) scaffold, **e** G1 (2:1) scaffold, **f** PLGA scaffold, **g** G2 (1:0) scaffold, **h** G2 (0:1) scaffold, **i** G2 (1:1) scaffold, **j** G2 (1:2) scaffold, **k** G2 (2:1) scaffold, and **l** PDLLA scaffold

**Fig. 8:**
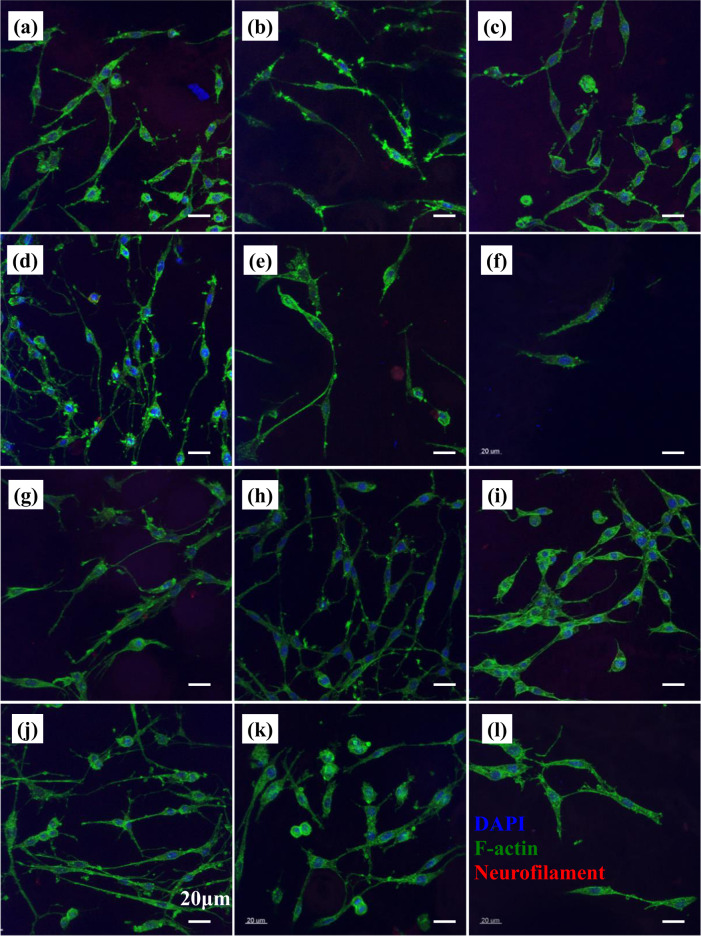
Morphology of PC12 cells cultured on different scaffolds as revealed under a confocal fluorescent microscope at day 7. **a** G1 (1:0) scaffold, **b** G1 (0:1) scaffold, **c** G1 (1:1) scaffold, **d** G1 (1:2) scaffold, **e** G1 (2:1) scaffold, **f** PLGA scaffold, **g** G2 (1:0) scaffold, **h** G2 (0:1) scaffold, **i** G2 (1:1) scaffold, **j** G2 (1:2) scaffold, **k** G2 (2:1) scaffold, and **l** PDLLA scaffold

The morphology of PC12 cells cultured on different scaffolds for 1, 4, and 7 days were investigated by CLSM and are shown in Figs. 6–8.

After 1-day culture, PC12 cells randomly scattered on scaffolds with low cell density. Cells on PLGA scaffolds (Fig. 6f) exhibited better cell attachment and cell spreading as compared to those on PDLLA scaffolds (Fig. 6l). Most of the cells on PDLLA scaffolds were in spherical shape with little or no neurite sprouting. A few cells on PLGA scaffolds exhibited elongated elliptical shape and initiated neurite sprouting and extension. Improved cell attachment and cell spreading on scaffolds in G1 group (Fig. 6a–e) were observed as compared to that in PDLLA scaffolds. More cells initiating neurite sprouting and exhibiting neurite elongation were found on scaffolds in G1 group except G1 (1:2) scaffolds as compared with both PLGA and PDLLA scaffolds. Neurite sprouting and elongation of PC12 cells were hardly noticed on G1 (1:2) scaffolds. The cell attachment and cell spreading on scaffolds with different component ratios of GDNF/PLGA fibers to NGF/PDLLA fibers in G2 group (Fig. 6g–k) were generally much enhanced as compared to scaffolds with corresponding component ratios of GDNF/PLGA fibers to NGF/PDLLA fibers in G1 group. More cells bearing neurite sprouting and neurite elongation were observed on G2 group scaffolds as compared to their counterpart scaffolds with corresponding component ratios of GDNF/PLGA fibers to NGF/PDLLA fibers in G1 group. However, neurite sprouting and elongation of PC12 cells were hardly observed on G2 (1:2) scaffolds, which was similar with cell morphology on G1 (1:2) scaffolds. Few cells with neurite branching were also noticed on G1 (1:0), G2 (1:0), G2 (0:1), and PLGA scaffolds. However, no neurofilament signal was detected in all scaffolds.

After 4-day culture, PC12 cells randomly distributed on scaffolds with medium cell density as shown in Fig. 7. Cells on different scaffolds exhibited much improved cell adhesion and spreading, neurite outgrowth, and neurite elongation at day 4 as compared to their morphology at day 1. Relatively weak intracellular neurofilament signals were detected in cells on different scaffolds. Cells on PLGA scaffolds (Fig. 7f) exhibited better cell attachment and cell spreading as compared to those on PDLLA scaffold (Fig. 7l). Most of the cells on PDLLA scaffolds were in elongated elliptical shape with neurite protrusions. Cells with elongated morphology and other irregular shapes spread randomly on PLGA scaffolds. Improved neurite sprouting, branching, and extensions were observed in PLGA scaffolds as compared to PDLLA scaffolds. With the stimulation of GDNF and/or NGF, more cells characterized by neurite sprouting, branching, and extensions were seen on growth factor-containing scaffolds (Fig. 7g–l). It was found that neurites extended with scarcely any sprouting and branching in both directions on G2 (0:1) scaffold (Fig. 7h), while neurites protruded randomly with much sprouting and branching on G2 (1:0) scaffold (Fig. 7g). With the increase of component ratio of GDNF/PLGA fibers to NGF/PDLLA fibers in different scaffolds, the phenomenon of neurite sprouting and branching was more obvious. It was worth mentioning that much neurite sprouting, branching, and elongations of PC12 cells occurred on G2 (1:2) scaffolds (Fig. 7j) at day 4, which was dramatically different from cell morphology on G2 (1:2) scaffolds at day 1. Similar phenomena were observed in cells on G1 scaffolds.

After 7-day culture, PC12 cells randomly spread on scaffolds with relatively high cell density and their morphology are shown in Fig. 8. Cells on different scaffolds exhibited further improved cell adhesion and spreading, neurite outgrowth, and neurite elongation at day 7 as compared to their morphology at day 4. Stronger intracellular neurofilament signals were detected in cells on different scaffolds. Cells were in elongated elliptical shape characterized by partial neurite extensions on both PLGA scaffolds (Fig. 8f) and PDLLA scaffolds (Fig. 8l). With the stimulation of GDNF and/or NGF, a large portion of cells characterized by significant neurite extensions appeared on growth factor-containing scaffolds (Fig. 8a–e, g–k). In general, the extent of neurite elongations and branching of PC12 cells was larger on scaffolds with different component ratios of GDNF/PLGA fibers to NGF/PDLLA fibers in G2 group as compared to scaffolds with corresponding fiber component ratios of GDNF/PLGA fibers to NGF/PDLLA fibers in G1 group. More cells characterized with neurite extensions and branching were observed on NGF/PDLLA scaffolds (Fig. 8b, h) as compared to GDNF/PLGA scaffolds (Fig. 8a, g) in both G1 and G2 group. The neurite extensions of cells on NGF/PLGA scaffold appeared to be larger as compared to that on GDNF/PLGA scaffolds in G1 group. However, the neurite length of cells on NGF/PLGA scaffolds appeared to be shorter as compared to that on GDNF/PLGA scaffolds in G2 group. Cells on G1 (1:2) scaffolds and G2 (1:2) scaffolds both exhibited most neurite outgrowth, largest neurite extensions, and also most distinct neurite branching as compared to other scaffolds in G1 group and G2 group, respectively, forming a partially interconnected neurite network. Interestingly, scaffolds composed of other component ratios (1:1 and 2:1) of GDNF/PLGA fibers to NGF/PDLLA fibers induced significantly less neurite outgrowth and shorter neurite extension in both G1 and G2 group.

### Quantification of neurite length and cell differentiation

**Fig. 9:**
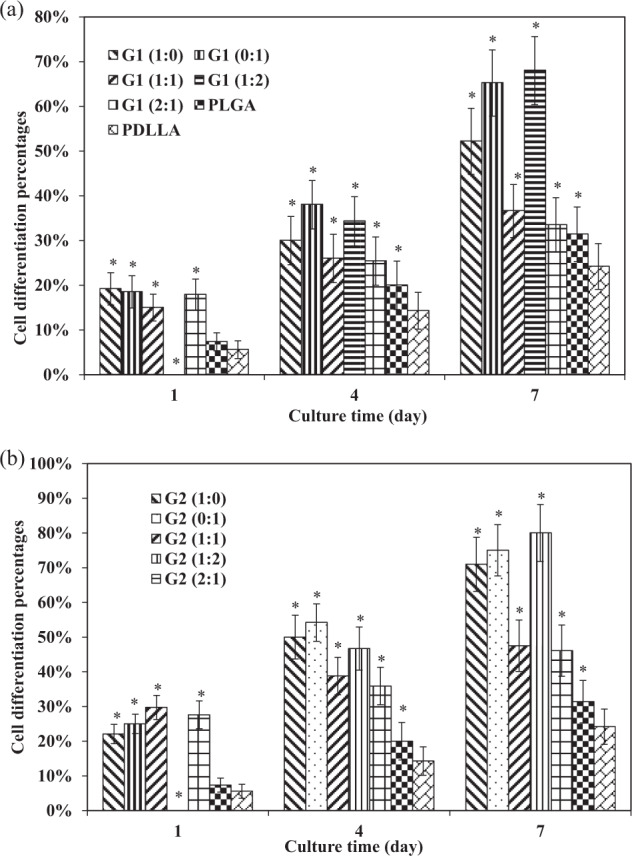
Cell differentiation of PC12 cells cultured on different scaffolds at days 1, 4, and 7. **a** G1 group, **b** G2 group. Cell differentiation statistically different than that in PDLLA control group. *p < 0.05

**Fig. 10:**
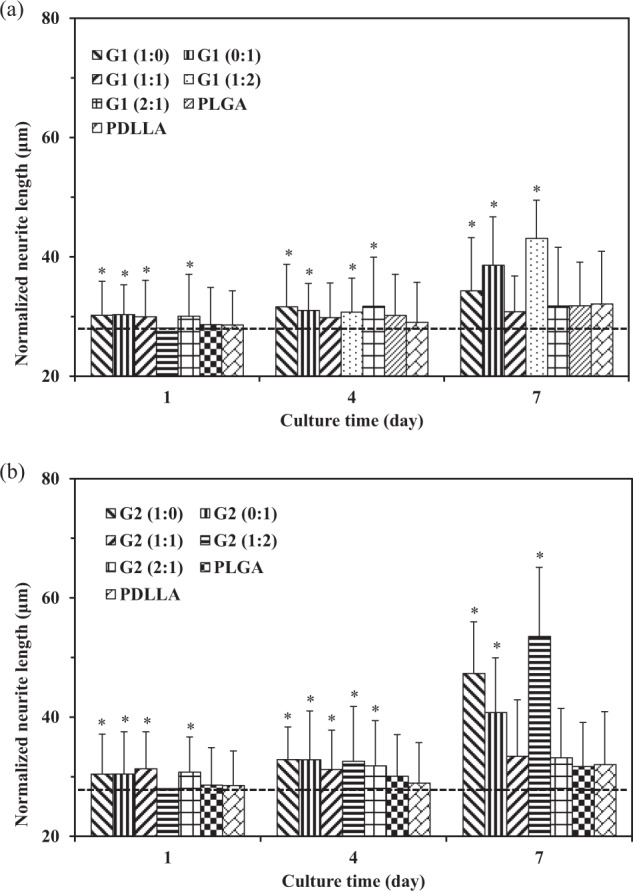
Normalized neurite length of PC12 cells cultured on different scaffolds at days 1, 4, and 7. **a** G1 group and PLGA, PDLLA control group, **b** G2 group and PLGA, PDLLA control group. Neurite protrusion statistically different than that in PDLLA control group. *p < 0.05

In order to better quantify the cell responses to different scaffolds, normalized neurite length and cell differentiation were determined that are shown in Figs. 9 and 10. The dotted line inserts in Fig. 10 represent neurite length at the level of 28 μm.

At day 1, it was found that cell differentiation percentages in scaffolds in G2 group were generally higher as compared to those scaffolds with corresponding fiber component ratios of GDNF/PLGA fibers to NGF/PDLLA fibers in G1 group. The degree of cell differentiation in PLGA scaffolds and PDLLA scaffolds was much lower as compared with all growth factor-containing scaffolds except G1 (1:2) and G1 (1:2) scaffolds.

The results of normalized neurite length showed that very limited neurite outgrowth and cell differentiation were induced by PLGA and PDLLA fibrous scaffolds at day 1. The delivery of growth factors from most of the scaffolds in G1 and G2 groups improved neurite outgrowth and neural differentiation to different extents.

At day 4, cell differentiation percentages in scaffolds in G2 group were higher as compared to those scaffolds with corresponding fiber component ratios of GDNF/PLGA fibers to NGF/PDLLA fibers in G1 group. The degree of cell differentiation in PLGA scaffolds and PDLLA scaffolds was much lower as compared with all growth factor-containing scaffolds. Higher degree of cell differentiation was observed in NGF/PDLLA scaffolds as compared to GDNF/PLGA scaffolds in both G1 and G2 groups. Bicomponent scaffolds with 1:1 and 2:1 fiber component ratios of GDNF/PLGA fibers to NGF/PDLLA fibers induced much lower cell differentiation as compared to monocomponent scaffolds in both G1 and G2 groups. It was worth mentioning that G1 (1:2) scaffolds and G2 (1:2) scaffolds induced the largest increase of cell differentiation percentage from day 1 to day 4. These results demonstrated that improved neural differentiation and neurite outgrowth was achieved at day 4.

At day 7, cell differentiation percentages in scaffolds in G2 group were higher as compared to those scaffolds with corresponding fiber component ratios of GDNF/PLGA fibers to NGF/PDLLA fibers in G1 group. The degree of cell differentiation in PLGA and PDLLA scaffolds was also much lower as compared with all growth factor-containing scaffolds. Again, higher degree of cell differentiation was observed in NGF/PDLLA scaffolds as compared to GDNF/PLGA scaffolds in both G1 and G2 groups. Bicomponent scaffolds with 1:1 and 2:1 fiber component ratios of GDNF/PLGA fibers to NGF/PDLLA fibers induced much lower cell differentiation as compared to monocomponent scaffolds in both G1 and G2 groups. However, bicomponent scaffolds with 1:2 fiber component ratios of GDNF/PLGA fibers to NGF/PDLLA fibers induced the highest cell differentiation in both G1 and G2 groups. Neurite length was much increased in G1 (1:0), G1 (0:1), G1 (1:2), G2 (1:0), G2 (0:1), and G2 (1:2) scaffolds, while only a slight increase in neurite length was observed in G1 (1:1), G1 (2:1), G2 (1:1), G2 (2:1), PLGA, and PDLLA scaffolds. The longest neurite outgrowth was obtained in bicomponent scaffolds with 1:2 fiber component ratios of GDNF/PLGA fibers to NGF/PDLLA fibers in both G1 and G2 groups. These results revealed that neural differentiation and neurite outgrowth was much improved to different extents and influenced by scaffold components at day 7.

### Morphology of cell-scaffold constructs

**Fig. 11:**
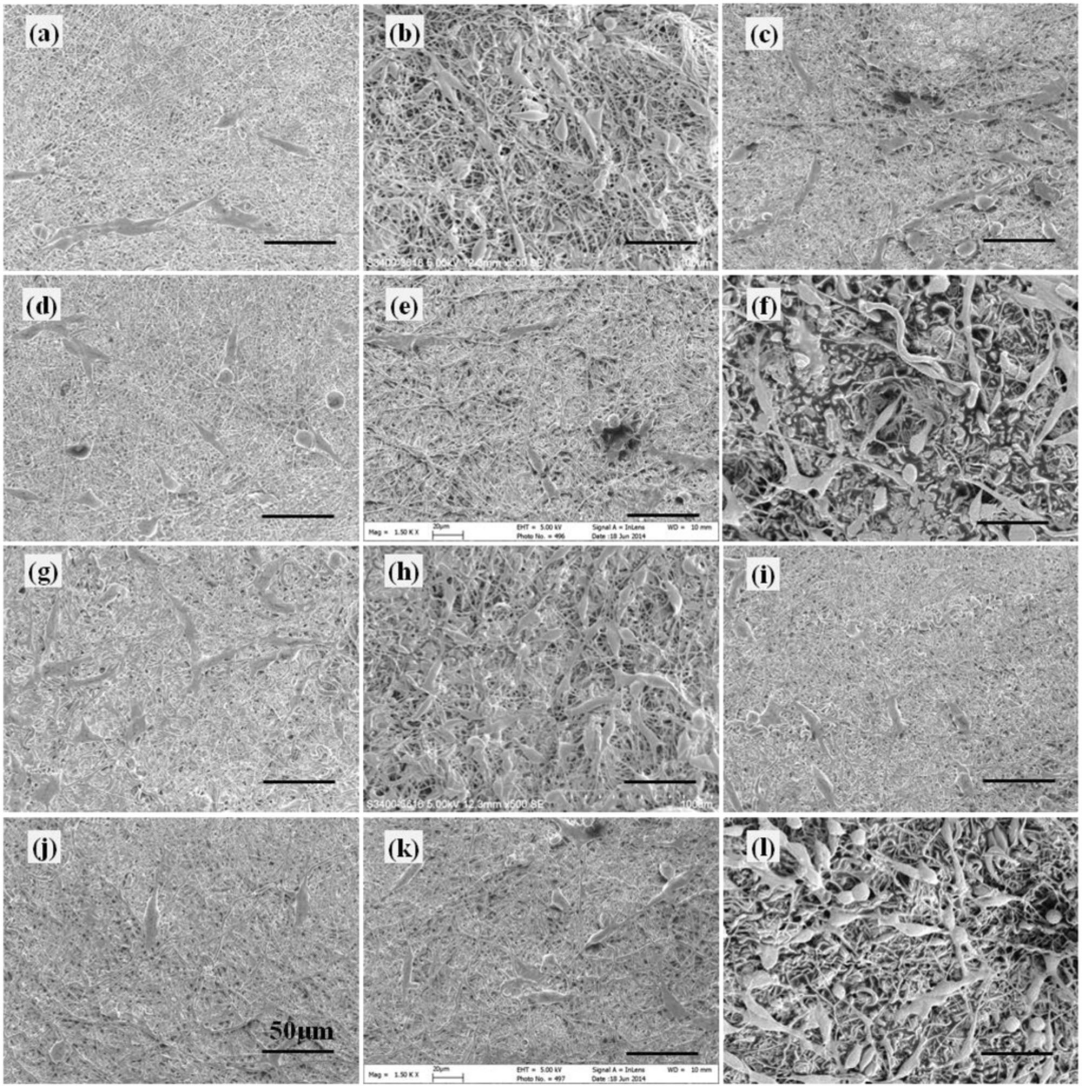
Morphology of PC12 cells cultured on different scaffolds under SEM at day 1. **a** G1 (1:0) scaffold, **b** G1 (0:1) scaffold, **c** G1 (1:1) scaffold, **d** G1 (1:2) scaffold, **e** G1 (2:1) scaffold, **f** PLGA scaffold, **g** G2 (1:0) scaffold, **h** G2 (0:1) scaffold, **i** G2 (1:1) scaffold, **j** G2 (1:2) scaffold, **k** G2 (2:1) scaffold, and **l** PDLLA scaffold

**Fig. 12:**
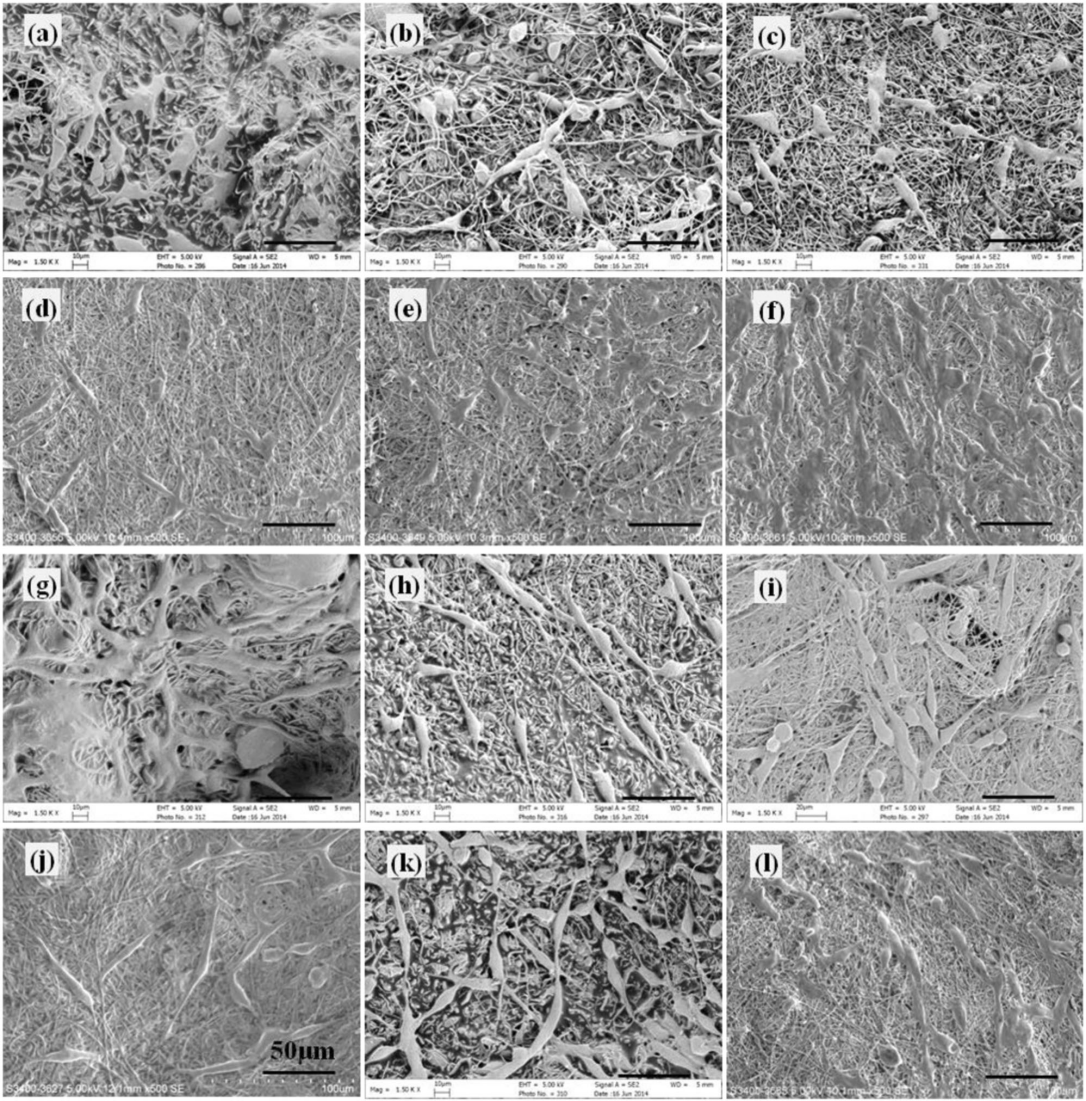
Morphology of PC12 cells cultured on different scaffolds under SEM at day 4. **a** G1 (1:0) scaffold, **b** G1 (0:1) scaffold, **c** G1 (1:1) scaffold, **d** G1 (1:2) scaffold, **e** G1 (2:1) scaffold, **f** PLGA scaffold, **g** G2 (1:0) scaffold, **h** G2 (0:1) scaffold, **i** G2 (1:1) scaffold, **j** G2 (1:2) scaffold, **k** G2 (2:1) scaffold, and **l** PDLLA scaffold

**Fig. 13:**
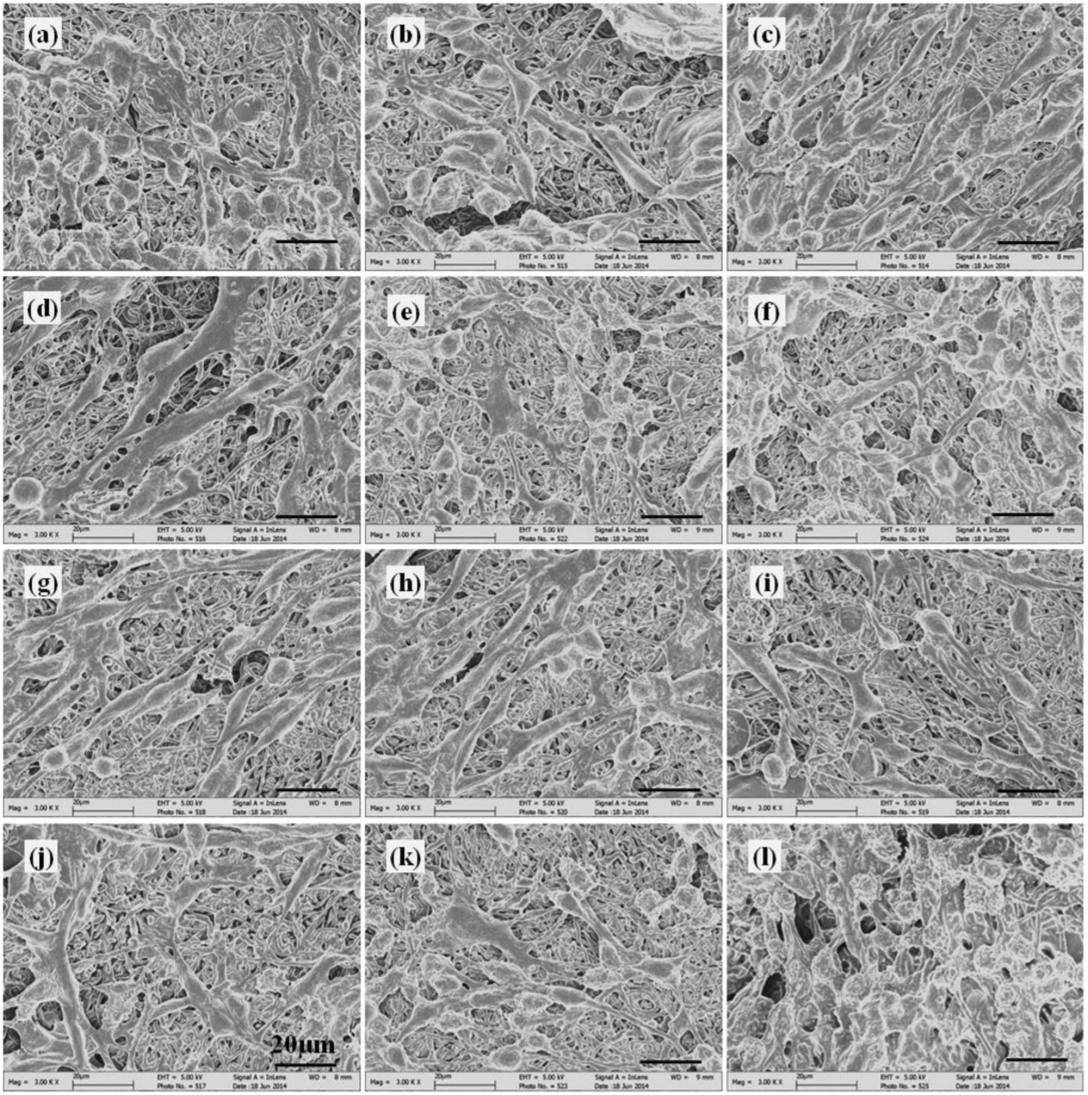
Morphology of PC12 cells cultured on different scaffolds under SEM at day 7. **a** G1 (1:0) scaffold, **b** G1 (0:1) scaffold, **c** G1 (1:1) scaffold, **d** G1 (1:2) scaffold, **e** G1 (2:1) scaffold, **f** PLGA scaffold, **g** G2 (1:0) scaffold, **h** G2 (0:1) scaffold, **i** G2 (1:1) scaffold, **j** G2 (1:2) scaffold, **k** G2 (2:1) scaffold, and **l** PDLLA scaffold

The morphology of different cell-scaffold constructs was examined under SEM and are shown in Figs. 11–13.

It was observed that at day 1, PC12 cells randomly scattered on scaffolds with low cell density. Cells on PLGA scaffolds (Fig. 11f) exhibited better cell attachment and cell spreading as compared to those on PDLLA scaffolds (Fig. 11l). Most of the cells on PDLLA scaffolds were in spherical shape with little or no neurite protrusions. A few cells on PLGA scaffolds exhibited elongated elliptical shape and initiated neurite extension. Improved cell attachment and cell spreading on scaffolds in G1 group (Fig. 11a–e) and G2 group (Fig. 11g–k) were observed as compared to that in PDLLA scaffolds. More cells initiating neurite sprouting and exhibiting neurite elongation were found on scaffolds in G1 group except G1 (1:2) scaffolds as compared with both PLGA and PDLLA scaffolds. More cells bearing neurite sprouting and neurite elongation were observed on G2 group scaffolds as compared to their counterpart scaffolds with corresponding component ratios of GDNF/PLGA fibers to NGF/PDLLA fibers in G1 group.

At day 4, cells adhered well and distributed randomly on scaffolds with medium cell density (Fig. 12). Scaffolds with fibrous structures were clearly observed underneath the cells. Most of the cells exhibited elongated elliptical shape and improved cell adhesion and spreading were found on GDNF/PLGA and PLGA scaffolds. Cells characterized by neurite protrusions were identified on all scaffolds. Some of the neurite protrusions appeared to extend along fibers underneath. More cells with neurite sprouting and neurite extensions were observed on growth factor-containing scaffolds, indicating that higher cell differentiation percentages were obtained in these scaffolds. The phenomenon of neurite outgrowth on scaffolds in G2 group was more evident as compared to scaffolds with corresponding component ratios of GDNF/PLGA fibers to NGF/PDLLA fibers in G1 group.

At day 7, cells proliferated well and covered a large portion of the surface of scaffolds (Fig. 13). Scaffolds maintained their fibrous structure underneath the cells. With the stimulation of GDNF and/or NGF, more cells characterized by neurite sprouting, branching, and extensions arose on growth factor-containing scaffolds. The phenomenon of neurite outgrowth on scaffolds in G2 group was more apparent as compared to scaffolds with corresponding component ratios of GDNF/PLGA fibers to NGF/PDLLA fibers in G1 group. In general, the morphological observations of different cell-scaffold constructs at days 1, 4, and 7 by SEM were consistent with the results obtained through CLSM.

## Discussion

Neurotrophic factors play important roles in neuronal survival, proliferation, differentiation, and nerve regeneration, independently or synergistically [1–4]. Different growth factor concentrations are required for different growth factors to generate biologically advantageous outcomes [5]. Sustained and local delivery of neurotrophic factors including GDNF and NGF have been employed to promote peripheral nerve regeneration recently [5, 6, 8, 16–18]. However, dual and sustained delivery of GDNF and NGF with different release kinetics, which may be very beneficial for enhanced peripheral nerve repair and functional recovery, is rarely investigated.

Electrospun fibers with fiber diameter ranging from nanometers to micrometers are efficient delivery vehicles for various therapeutic biomolecules owing to their high surface area to volume ratio. There is concern about bioactivity loss of biomolecules in electrospun fibers due to their potential deactivation and denaturation that might occur during solution preparation, electrospinning at high voltage, freeze-drying, and sample sterilization [19–21]. Quite a few reports demonstrated that protein released from electrospun scaffolds could induce various cellular responses, indicating that protein activity was at least partially preserved during electrospinning [22–24]. Emulsion electrospinning and coaxial electrospinning techniques could help preserve the bioactivity of vulnerable biomolecules and provide delivery of these biomolecules in a controlled manner [25, 26].

In the current investigation, GDNF and NGF were incorporated into robust PLGA and PDLLA fibers, respectively, through emulsion electrospinning (Fig. 1). The “evaporation and stretching-induced de-emulsification” mechanism helped elucidate the formation of core-shell structures [27]. Bicomponent fibrous scaffolds with different GDNF/PLGA fiber to NGF/PDLLA fiber component ratios and different fiber diameters were produced through DSDP-ES. The average fiber diameter in bicomponent scaffolds, which could be taken as a weighted average of fiber diameter of the two types of fibers (GDNF/PLGA fibers to NGF/PDLLA fibers), decreased proportionally with the increase of GDNF/PLGA fiber to NGF/PDLLA fiber ratio, suggesting that the scaffolds had a controllable composition as expected. The strategy of utilizing controlled deposition of fibers to successfully produce bicomponent electrospun scaffolds with tunable composition was also reported [13, 28].

In this investigation, by incorporating growth factors into polymer matrix with different degradation rates and tuning component ratios in bicomponent scaffolds through DSDP-ES, sustained and concurrent release of GDNF and NGF from fibrous bicomponent scaffolds was achieved. Tunable cumulative release of GDNF and NGF was also obtained by varying GDNF/PLGA fiber to NGF/PDLLA fiber component ratio and fiber diameter in electrospun scaffolds. Following an initial burst release in the first 24 h, sustained release of NGF and/or GDNF from corresponding fibers was found during the release period. The initial burst release was attributed to the growth factor-contained minute reservoirs formed on or close to the surface of fibers during emulsion electrospinning. During emulsion electrospinning, the majority of stretched water droplets moved inward coalesced in the central region and formed the water phase core. However, a minority of water droplets were detained, while the outer layer solidified due to rapid evaporation of organic solvent. These tiny water droplets dwelling on or near the surface of fibers served as reservoir of growth factors and rendered initial burst release through diffusion when the scaffolds were immersed in testing liquid. After the initial burst release, sustained release was obtained that was attributed to either diffusion of growth factor from interior of fibers to immersion liquid through the polymer matrix of fibers or a combination of diffusion and polymer matrix degradation.

The cumulative release amount of NGF decreased proportionally with the increase of GDNF/PLGA fiber to NGF/PDLLA fiber component ratio in both G1 and G2 groups. Scaffolds with thicker fibers (G1 group) alleviated initial burst release and decreased the release rate of NGF comparing with those with thinner fibers (G2 group). It was also noticed that, at later stage of release tests, release rate of NGF was slightly accelerated in bicomponent scaffolds as compared with that in monocomponent scaffold.

The cumulative release profile of GDNF showed that release of GDNF from bicomponent scaffolds was marginally hindered in comparison with that from their monocomponent counterpart. The slower GDNF release was raised with the increase of PDLLA component in bicomponent scaffolds. More interestingly, a biphasic release profile of GDNF was noticed in G1 (1:0) scaffolds. Following the initial burst release, a sustained release was achieved that may be attributed to diffusion. A remarkable increase of release rate was noticed at around 2 weeks, which was very likely caused by the fusion and erosion of polymer matrix. SEM images of scaffolds at different release periods were employed to help investigate the release mechanism.

Comparing the release profiles of growth factors, it could be seen that biomedical polymers with different wettability and degradation rates could be leveraged to tailor the release behavior of incorporated growth factor. NGF and GDNF were herein incorporated into PDLLA fibers and PLGA fibers, respectively, to obtain different release profile as PLGA was less hydrophobic and had much faster degradation rate than PDLLA [14]. The release rate of GDNF was therefore much faster than that of NGF. Average diameter of electrospun fibers in scaffolds was also a useful control variable to manipulate the release profile of growth factors including the initial burst release and subsequent sustained release. It could not be ignored that in bicomponent scaffolds, the release behavior of either growth factor might be affected to some degree by its counterpart component. It was speculated that hydrophilicity/hydrophobicity of two types of polymer matrix resulted in the marginal fluctuation of individual release profile in bicomponent scaffolds. The release of hydrophilic NGF through bicomponent scaffolds was upregulated by less hydrophobic PLGA component; in contrast, the release of hydrophilic GDNF was downregulated by more hydrophobic PDLLA component.

The electrospun fibers in scaffolds had a distribution of fiber diameters and both types of fibers underwent fiber recoiling, swelling, fusion, and minor erosion as release test proceeded. Both diffusion and matrix erosion contributed to release kinetics of protein from biodegradable polymer matrix such as PLGA and PDLLA. Therefore, the release behavior of GFs from electrospun scaffolds could hardly be explained by any simple or generalized model. Although there is no perfect model for modeling the release behavior of NGF or GDNF from bicomponent scaffolds, the results in this study did demonstrate that concurrent and sustained release of NGF and GDNF with tunable amounts and ratios could be achieved.

The influences of these electrospun fibrous scaffolds providing delivery of GDNF and/or NGF with different release kinetics on cell proliferation and neuronal differentiation were investigated using PC12 cells. Biocompatibility is an essential character of tissue engineering scaffolds. Cell proliferation on different scaffolds was investigated by MTT method (Fig. 5). Although cells proliferated significantly from day 1 to day 7 on all scaffolds, different proliferation rates were obtained for different scaffolds. The cell proliferation on growth factor-containing scaffolds may not be as indicative for scaffold cytotoxicity as in experiments where other types of cells were used. The cell responses to scaffolds providing growth factor stimulation were actually a dynamic equilibrium between cell proliferation and differentiation as PC12 cells responded to neurotrophic factors including NGF and GDNF and differentiated into neuron-like phenotypes characterized by halted proliferation and neurite outgrowth [12]. No or negligible cytotoxicity was found for PLGA and PDLLA fibrous scaffolds as both PLGA and PDLLA were biocompatible and biodegradable materials. As no other materials except growth factors were introduced in growth factor-containing scaffolds as compared to PLGA and PDLLA scaffolds, it may be assumed that these growth factor-containing scaffolds also exhibited little or no cytotoxicity.

To test the hypothesis that dual and sustained delivery of GDNF and NGF with different release kinetics can modulate neural differentiation, the morphology of PC12 cells cultured on different scaffolds was investigated and neurite length was measured for determining neural differentiation percentages. Fibrous structures of electrospun scaffolds were maintained (Figs. 11–13). Proliferation or infiltration of PC12 cells into scaffold interior was not commonly observed even though in some scaffolds cells were seen under the surface fibers of scaffolds. Improved cell adhesion and spreading were noticed on PLGA scaffolds as compared to PDLLA scaffolds, which might be attributed to different surface wettability of two types of scaffolds. PLGA fibrous scaffolds had much improved wettability as compared to PDLLA fibrous scaffolds due to hydrophilic glycolic acid segments in PLGA polymer. It is known that surface wettability plays an important role in cell-scaffold interaction including cell adhesion and proliferation. It was reported that an increase in wettability of electrospun fibrous scaffolds increased endothelial cell spreading but decreased cell adhesion [29].

A small portion of PC12 cells differentiated into neuron-like phenotypes characterized by neurite outgrowth along electrospun fibers on both PLGA and PDLLA scaffolds, suggesting that fibrous topography might induce neurite outgrowth and neural differentiation to some extent. This result was consistent with recent studies by other groups that utilized surface topography to enhance cell neural commitment through a process requiring focal adhesion [30–34].

Stimulation of PC12 cells with GDNF and/or NGF released from various scaffolds induced much improved neurite outgrowth and neural differentiation. In general, higher amounts of growth factors induced more cell differentiation and longer neurite extensions. However, the situation of neurite outgrowth and neural differentiation on different growth factor-containing scaffolds varied from one type of scaffold to another. GDNF and NGF released from GDNF/PLGA scaffolds and NGF/PDLLA scaffolds, respectively, both triggered neurite extensions independently. GDNF and NGF released from bicomponent scaffolds with 1:2 component ratio of GDNF/PLGA fibers to NGF/PDLLA fibers induced no neural differentiation at day 1, but much more neural differentiation at day 4 and highest neural differentiation at day 7. In contrast, GDNF and NGF released from bicomponent scaffolds with 1:1 and 2:1 component ratios of GDNF/PLGA fibers to NGF/PDLLA fibers induced much less neural differentiation at both day 4 and day 7. It appeared that GDNF and NGF released from G1 (1:2) and G2 (1:2) scaffolds exerted a synergistic effect on promoting neural differentiation, while GDNF and NGF released from other bicomponent scaffolds did not. This phenomenon might be attributed to neurotrophic factor dose-dependent neurite outgrowth and signaling pathways of GDNF and NGF [5, 12, 35].

Neurite outgrowth is neurotrophic factor dose-dependent with optimal concentrations in the range of 1–10 ng/mL as low amounts of NGF are preferred for optimal neurite outgrowth but high amounts of GDNF are favorable for maximum neurite outgrowth [34]. NGF and GDNF stimulate neurite extension through the same mitogen-activated protein kinase/extracellular signal-regulated kinase pathway but by binding to their respective tyrosine kinase receptors TrkA and ReT [5, 12]. Both NGF and GDNF can upregulate the expression of their respective receptors and combined NGF/GDNF stimulation can upregulate the expression of two receptors simultaneously, which might result in the observed synergistic effect of neural differentiation on G1 (1:2) and G2 (1:2) scaffolds. However, the molecular mechanisms of the communication pathways among GDNF, NGF, ReT, and TrkA remain further exploration.

## Conclusions

In this study, fibrous bicomponent scaffolds for the dual delivery of growth factors were successfully produced through emulsion electrospinning and DSDP-ES. GDNF and NGF were incorporated into core-shell structured PLGA and PDLLA nanofibers, respectively. Concurrent and sustained release of GDNF and NGF from bicomponent scaffolds was achieved and tunable release profiles were obtained. All of the produced electrospun fibrous scaffolds exhibited no or negligible cytotoxicity. Stimulation of growth factors released from electrospun scaffolds could induce much improved neurite outgrowth and neural differentiation. GDNF and NGF released from scaffolds could induce dose-dependent neurite extensions independently. Dual delivery of neurotrophic factors from bicomponent scaffolds exerted a synergistic effect on promoting neural differentiation, which might be promising for peripheral nerve regeneration.
